# How many people will need palliative care in 2040? Past trends, future projections and implications for services

**DOI:** 10.1186/s12916-017-0860-2

**Published:** 2017-05-18

**Authors:** S. N. Etkind, A. E. Bone, B. Gomes, N. Lovell, C. J. Evans, I. J. Higginson, F. E. M. Murtagh

**Affiliations:** 1 King’s College London, Cicely Saunders Institute, Department of Palliative Care, Policy and Rehabilitation, London, UK; 2 University of Coimbra, Faculty of Medicine, Coimbra, Portugal; 3 Sussex Community NHS Foundation Trust, Brighton, UK

**Keywords:** Mortality, Forecasting, Palliative care, Needs assessment, Health services needs and demand, Chronic disease, Comorbidity

## Abstract

**Background:**

Current estimates suggest that approximately 75% of people approaching the end-of-life may benefit from palliative care. The growing numbers of older people and increasing prevalence of chronic illness in many countries mean that more people may benefit from palliative care in the future, but this has not been quantified. The present study aims to estimate future population palliative care need in two high-income countries.

**Methods:**

We used mortality statistics for England and Wales from 2006 to 2014. Building on previous diagnosis-based approaches, we calculated age- and sex-specific proportions of deaths from defined chronic progressive illnesses to estimate the prevalence of palliative care need in the population. We calculated annual change over the 9-year period. Using explicit assumptions about change in disease prevalence over time, and official mortality forecasts, we modelled palliative care need up to 2040. We also undertook separate projections for dementia, cancer and organ failure.

**Results:**

By 2040, annual deaths in England and Wales are projected to rise by 25.4% (from 501,424 in 2014 to 628,659). If age- and sex-specific proportions with palliative care needs remain the same as in 2014, the number of people requiring palliative care will grow by 25.0% (from 375,398 to 469,305 people/year). However, if the upward trend observed from 2006 to 2014 continues, the increase will be of 42.4% (161,842 more people/year, total 537,240). In addition, disease-specific projections show that dementia (increase from 59,199 to 219,409 deaths/year by 2040) and cancer (increase from 143,638 to 208,636 deaths by 2040) will be the main drivers of increased need.

**Conclusions:**

If recent mortality trends continue, 160,000 more people in England and Wales will need palliative care by 2040. Healthcare systems must now start to adapt to the age-related growth in deaths from chronic illness, by focusing on integration and boosting of palliative care across health and social care disciplines. Countries with similar demographic and disease changes will likely experience comparable rises in need.

**Electronic supplementary material:**

The online version of this article (doi:10.1186/s12916-017-0860-2) contains supplementary material, which is available to authorized users.

## Background

Many people approaching the end-of-life could benefit from palliative care, which can be defined as a patient-centred approach to care in advanced disease, focusing on quality of life and relief of symptoms [1–6]. The way in which palliative care is delivered depends on individual patient needs. Palliative care is frequently delivered by non-specialists, for example, as advance care planning in a primary care setting or symptom management in secondary care. Specialist multidisciplinary palliative care teams deliver care for more complex needs in a variety of settings, including hospitals, hospices and in the community, although they can also provide more straightforward end-of-life care.

More palliative care support is likely to be required for some illnesses and clinical syndromes, such as multimorbidity, chronic progressive illnesses with long disease courses, and diseases with complex symptoms. This is important because, alongside demographic changes [7, 8], the incidence, prevalence and mortality of chronic illness is rising worldwide [8–10], with an increase in long-term conditions and multimorbidity [9, 10].

Healthcare services will therefore need to adapt to provide appropriate services for changing populations, and this will require more resources, including for palliative and end-of-life care. Estimates of the cost of care in the last year of life are consistently high [11–13], and in England, these costs are expected to grow by 25% by 2030 [14].

However, despite the growth in drivers of palliative care needs, access to palliative care remains inconsistent, even in high-income countries, with the number of providers varying from 5 to 680 per million population [12]. Overall, only a minority who need palliative care, perhaps as low as 14%, receive it [12, 15–17].

To improve access to palliative care, we must first investigate and understand the level of need in the population. A helpful definition of need is that of Stevens and Raftery, who define healthcare need as the ‘ability to benefit’ from healthcare services [18–20]. This definition can be applied to individual health problems, and also to population health. It is thus informative for epidemiological research and health service planning.

Current population needs for palliative care have been estimated in several countries using different methods for its measurement [21, 22], based on patient diagnoses [22–24], symptom prevalence [25] and population surveys [5, 26]. Most of these methods produce broadly similar results in terms of how many people are estimated to need palliative care [5, 22, 24–26], although additional data sources can increase sensitivity [22, 27].

However, in light of the major demographic and disease changes occurring and expected to occur globally, we need to know more than the current level of palliative care need – we need to know how the healthcare needs of people at the end of life will change over time in order to plan healthcare services. Projections of palliative care need are therefore required to guide service development. In this study, we aim to estimate the numbers and proportions of people at the end-of-life stage who may benefit from palliative care, and project this estimate of palliative care need onto future populations, using two high-income countries, namely England and Wales, as examples.

## Methods

### Design

Secondary analysis of routinely available national death registry and population data (both provided by the Office for National Statistics (ONS)) for England and Wales. We estimated palliative care need for the time period of 2006 to 2014 using two estimates, namely an estimate of palliative care need at 75% of all deaths, and an estimate based on a set of relevant causes of death. We then projected these estimates of palliative care need using official mortality forecasts to model estimates of palliative care need up to 2040. We developed these projections by investigating mortality in specific disease groups, the effect of age and incorporating an estimate of pain prevalence.

### Data sources

#### Death registry data (2006–2014)

As part of their Deaths Registered series, the ONS records the ‘main’ cause of death for every death registered in each calendar year in England and Wales using the information supplied on the death certificate. We used this data to identify causes of death for inclusion in our palliative care estimate. This approach has limitations, as it relies on accurate death certification. However, evidence suggests that, at a population level, cause of death correlates well to known diagnoses [28]. The Deaths Registered series uses an International Classification of Disease (ICD)-10 code-based classification, which was last updated in 2011 (Additional file 1) [29].

#### Anticipated population and mortality changes (2014–2040)

Population data are collected by the ONS using a mid-year estimate on June 30. The 2014 data used in this analysis is based on 2010 Census data with subsequent annual adjustments. The ONS uses these population data, along with assumptions about fertility, mortality and migration, to develop population and mortality projections. Assumptions are based on long-term population trends. For this analysis, we used the mortality projections from the principal population projection for England and Wales up to 2040. We limited our projection to 2040 because, after this, there is increased uncertainty in mortality assumptions [7].

Mortality and projections data for England and Wales are produced together using uniform methodology; therefore, data for both countries can be easily combined. More detail on technical aspects of combining these datasets can be found in Additional file 1.

### Estimates of current palliative care need

We used two estimates of palliative care need, both of which are based on a proportion of population deaths as palliative care needs are most evident at the end of life. This has served as a reasonable proxy for population palliative care need in previous population analyses [22, 24].

#### Estimate 1

The first estimate, based on population survey by Gomez-Batiste et al. [26], assumes that approximately 75% of people in high-income countries die from chronic progressive diseases with evolving and increasing healthcare needs, and that, therefore, 75% of all people at the end-of-life stage may benefit from specialist or non-specialist palliative care.

#### Estimate 2

The second estimate is calculated based on deaths from specific chronic diseases. We chose a diagnosis-based estimate because we hypothesised that changes in the prevalence of chronic illnesses would be a key factor affecting the level of need. For this estimate, we considered that people dying of chronic illnesses are very likely to have complex physical, psychological, spiritual and/or social problems that can be improved by either specialist or non-specialist palliative care services. They therefore have the ability to benefit from palliative care, and hence have palliative care needs as per the Stevens and Raftery definition [18, 19]. At a population level, palliative care need can therefore be estimated as the proportion of people who die from chronic diseases.

High quality, routinely collected data to support this estimate are publicly available. We used ICD-10 diagnostic codes previously used to estimate population palliative care need (Table 1) [21, 22], and defined a person requiring palliative care as someone dying from an illness with an included ICD-10 code [22]. We calculated the percentage of deaths from these codes out of all deaths in a calendar year, which gave our prevalence estimate of palliative care need.

**Table 1:** International Classification of Disease-10 codes used to estimate palliative care need [22]

Grouping	Code	Conditions included
Cancer	C00–C97	All deaths from malignant neoplasms
Organ failure	I00–I52 (excl. I12 & I13) J40–J47, J96 I12, I13, N17, N18, N28 K70–K77	Heart disease and heart failure Chronic lower respiratory disease, respiratory failure Reno-vascular disease, renal failure Liver disease
Dementia	F01, F03, G30, R54	Dementia, vascular dementia, Alzheimer’s disease, senility
Other	G10, G12.2, G20, G23.1, G35, G90.3 I60–I69 B20–B24	Huntington’s disease, motor neurone disease, Parkinson’s disease, progressive supranuclear palsy, multiple sclerosis, multi system atrophy Haemorrhagic, ischaemic and unspecified stroke HIV

### Projections of palliative care need estimates

#### Projection methodology

Projection method 1: We calculated palliative care need estimate 1 according to 75% of deaths for the most recent available year, 2014. We then applied this estimate to ONS mortality projections up to 2040, assuming the proportion of deaths requiring palliative care would remain constant.

Projection method 2 (assuming proportion of palliative care needs constant): We calculated palliative care need estimates according to age- and sex-specific deaths from included ICD-10 codes for 2014 (estimate 2), and applied this estimate to mortality projections up to 2040, assuming the proportion would remain constant as per projection method 1.

Projection method 2 (assuming an annual change): It is unlikely in reality that the prevalence of chronic disease, and hence the proportion of people dying in need of palliative care, will remain constant over time. We therefore refined projection method 2 to project a measure of annual change in palliative care need. Using the ICD-10 code based cause-of-death estimate, we calculated the proportion of deaths with palliative care need using the cause of death estimate, from 2006 to 2014. We then calculated a mean annual change in the proportion of deaths requiring palliative care over this base period. We assumed the annual change would continue to occur in a linear fashion, and applied the resulting proportion to population forecasts up to 2040. To account for changes in coding of death registry data which occurred in 2011 [29], we calculated mean annual change based on two base periods (2006 to 2014 and 2011 to 2014). See Additional file 1 for further details.

#### Projecting palliative care need by age group and disease group

Using projection method 2 (assuming annual change) as a basis, we developed the projections to investigate projected changes in palliative care need in specific age and disease groups. We first described the number of people in each 5-year age group who are projected to need palliative care between 2014 and 2040, and then undertook disease group-specific projections.

For disease group-specific projections, we categorised our ICD-10 codes into four groups, namely deaths from cancer, dementia, organ failure (including cardiac, respiratory, renal and liver disease) and other included diseases (neurological disease, stroke and HIV). We separated cerebrovascular disease and dementia to demonstrate the impact of changes in dementia deaths.

#### Incorporating pain prevalence

Symptom prevalence is key to palliative care need and highlights the complexity of need. It has been accounted for in assessments of population palliative care need [25]. From a literature review, we identified estimates of the prevalence of pain in the last year of life. Pain prevalence in cancer [25, 30–32], organ failure [31, 32], dementia [33, 34] and neurological conditions [35] has been estimated. Based on this, we applied pain prevalence estimates onto our disease group projections to provide the estimated number of people dying from included diseases who also suffered from pain.

### Sensitivity analyses

We undertook sensitivity analyses to assess the robustness of our methodology. Firstly, we adjusted for the time-period over which death registry and mortality projections data are collected and assessed the effect this had on our projections. Secondly, we applied the Lee–Carter approach to mortality projections to produce an alternative projection, and compared this to our main methodology [36]. See Additional files 1, 2 and 3 for further details.

### Ethical approval

Since this study used routinely collected anonymised, publicly available data, no ethical approvals were needed.

## Results

### Estimates of palliative care need from 2006 to 2014

Between 2006 and 2014, the number of deaths in England and Wales has remained relatively constant, changing from 502,599 to 501,424. In this time period, population palliative care need, based on the ICD-10 code estimate, has also risen from 364,283 (72.5% of deaths) to 375,398 (74.9%; mean annual change of 0.30%). Cancer deaths have increased from 135,635 to 143,638; organ failure deaths reduced from 145,604 to 129,338; dementia deaths increased from 27,364 to 59,199; and other deaths reduced from 55,680 to 43,223. After the ICD-10 coding change, from 2011 to 2014, palliative care need increased from 357,251 (73.8%) to 375,398 (74.9%; mean annual change 0.37%).

### Anticipated population changes from 2014 to 2040

**Fig. 1:**
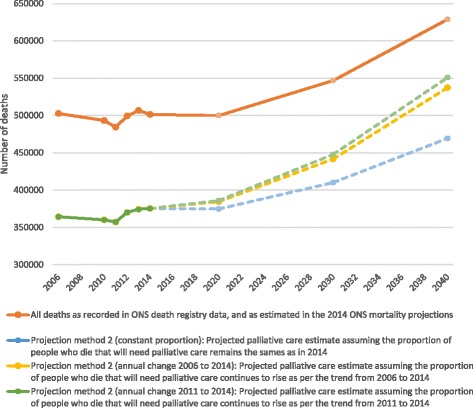
Estimated number of people requiring palliative care from 2006 to 2040. Projections of overall population palliative care need according to International Classification of Disease-10 estimates. Solid lines indicate estimates based on actual mortality data. Dotted lines indicate projection models. Note: projection method 1 is not presented in this figure, since it produces very similar results to projection method 2 (assuming proportion of palliative care needs constant). See also Table 3

Population changes: According to ONS forecasts, the population of England and Wales will grow by 9,443,000 between 2014 and 2040 (Table 2). The proportion of people aged over 65 will increase from 17.7% to 24.2%. These changes impact on the number of people expected to die in 2040. The ONS projects a rise in the number of deaths from 501,424 in 2014 to 628,659 in 2040 (127,234 (25.4%) increase). Additionally, more people are projected to die at an older age. The proportion of people dying over the age of 85 will rise from 38.8% in 2014 to 53.2% in 2040. Our projections take these changes into account (Table 3 and Fig. 1).

**Table 2:** England and Wales population forecasts and projected number of deaths, 2014–2040 [7]

	Population (‘000 s)				Deaths			
	2014	2020	2030	2040	2014	2020	2030	2040
All	57,409	60,011	63,763	66,852	501,424	499,868	546,768	628,659
0–44	32,707	33,498	34,662	35,231	18,703	17,229	15,335	13,496
45–64	14,550	15,228	15,127	15,441	60,482	59,229	52,641	44,589
65–74	5501	5972	6831	7168	82,418	83,373	77,863	77,056
75–84	3297	3744	4863	5744	145,366	141,247	160,314	159,091
85 +	1354	1569	2280	3268	194,455	198,790	240,603	334,427
% aged ≥ 65	17.7	18.8	21.9	24.2	84.2	84.7	87.6	90.8
% aged ≥ 85	2.4	2.6	3.6	4.8	38.8	39.8	44.0	53.2

**Table 3:** Estimated palliative care need in England and Wales, 2014–2040, using two different projection methods

		Deaths potentially requiring palliative care			
		2014	2020	2030	2040
All deaths		501,424	499,838	546,768	628,659
Projection method 1^a^	n (%)	376,068 (75.0)	374,879 (75.0)	410,076 (75.0)	471,494 (75.0)
Projection method 2 (constant proportion)^b^	n (%)	375,398 (74.9)	374,648 (75.0)	410,018 (75.0)	469,305 (74.9)
Projection method 2 (annual change 2006–2014)^c^	n (%)	375,398 (74.9)	384,343 (76.6)	441,625 (80.4)	537,240 (85.0)
Projection method 2 (annual change 2011–2014)^d^	n (%)	375,398 (74.9)	385,977 (77.3)	447,688 (82.6)	550,734 (87.1)

^a^Projection method 1: 75% of all deaths [5, 26]

^b^Projection method 2 (constant proportion: proportion of all deaths requiring palliative care according to included International Classification of Disease (ICD)-10 codes, assuming this proportion remains the same as in 2014

^c^Projection method 2 (annual change 2006–2014) projection of mean annual change in proportion needing palliative care according to ICD-10 estimate from 2006 to 2014

^d^Projection method 2 (annual change 2011–2014): projection of mean annual change in ICD-10 estimate from 2011 to 2014

### Projections of palliative care need from 2014 to 2040

Projection method 1: If the proportion of people who die needing palliative care remains at 75.0% of all deaths, 471,494 people (95,426 more) will need palliative care in 2040 (Table 3).

Projection method 2 (constant proportion): If age- and sex-specific proportions of deaths requiring palliative care, using the ICD-10 estimate, remain the same as in 2014, the number of people requiring palliative care will grow by 25.0% (from 375,398 to 469,305 people/year; 93,907 increase) by 2040 (Table 3 and Fig. 1).

Projection method 2 (assuming annual change): If the 2006–2014 age- and sex-specific trend in palliative care need according to the ICD-10 estimate continues, the proportion of people needing palliative care will rise to 85.5% of all deaths (537,240). This corresponds to 161,842 more people needing palliative care each year than in 2014. If the more recent 2011–2014 trend is used, 87.6% of all deaths (550,734; 175,336 more) will need palliative care by 2040.

### Projections of palliative care need from 2014 to 2040 by age and disease groups

#### Palliative care need by age group

**Fig. 2:**
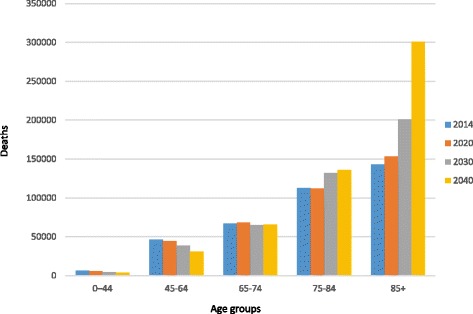
Number of people estimated to require palliative care by age, 2014–2040

Based on projection method 2 (assuming annual change), we found that the number of people who die aged 0–44 years who are likely to need palliative care will fall between 2014 and 2040 from 6465 to 3891. Similarly, palliative care need in the 45–69 age group will fall from 46,201 to 31,132 due to expected mortality improvements in this age group (Fig. 2). However, the number of people aged 85 and older who need palliative care will more than double, rising from 142,716 in 2014 (38.0% of overall palliative care need estimate) to 300,910 in 2040 (56.0% of the need estimate).

#### Palliative care need by disease group

**Fig. 3:**
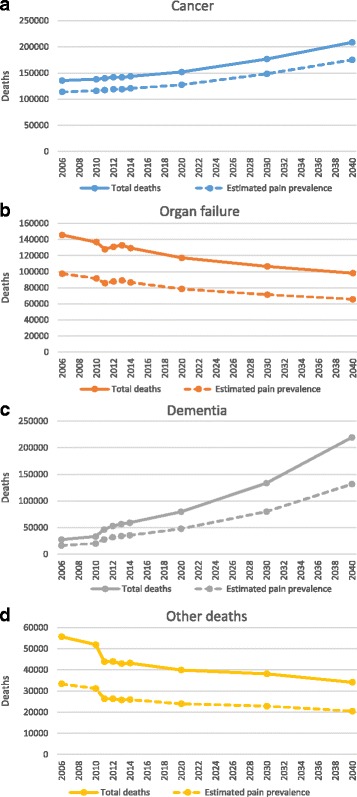
Number of people from the four disease groups estimated to require palliative care from 2006 to 2040. Solid lines represent total estimate, Dotted lines represent estimates of pain prevalence for each disease group. **a** Cancer deaths, **b** Organ failure deaths (chronic cardiovascular, respiratory, renal or liver disease), **c** Dementia deaths, and **d** Deaths from Other illnesses (degenerative neurological disease, cerebrovascular disease and HIV) that are likely to require palliative care between 2006 and 2040. The recent trend in deaths from each of these disease groups has been calculated and projected from 2014 to 2040. For **a** and **b** the trend from 2006 to 2014 has been projected. For **c** and **d** the trend from 2011 to 2014 has been projected (to avoid errors based on coding changes between 2010 and 2011)

Our disease group projections were based on projection method 2 (assuming annual change). Assuming that recent trends in mortality will continue in a linear fashion, cancer deaths will rise from 143,638 in 2014 to 208,636 deaths per year in 2040. Dementia deaths are projected to rise from 59,199 to 219,409. Organ failure deaths are projected to fall in people aged below 90, resulting in an overall reduction from 129,338 to 98,092. However, in people dying aged 90 and over, organ failure deaths are projected to rise by 12,088. Other deaths will fall from 43,223 to 34,134 (Fig. 3).

#### Projection incorporating pain prevalence

Our estimates of pain prevalence in the four groups of diseases were 84% in cancer [25], 67% in organ failure [25], and 60% in dementia and other [33]. Applying these pain prevalence estimates to refine the projection of palliative care need according to method 2 (assuming annual change 2006–2014) indicated that, by 2040, 393,101 (70.1%) people within the palliative care need estimate will have pain (Fig. 3); of these, 175,254 deaths from cancer, 131,645 from dementia, 65,721 from organ failure, and 20,480 from other diseases will have pain. This demonstrates that, whilst the raw numbers of people dying from dementia are projected to be higher than cancer by 2040, the number of people with cancer and pain (175,254) will be greater than the number of people with dementia and pain (131,645).

#### Sensitivity analyses

Our projection using the Lee–Carter approach [36] found that 551,146 people would need palliative care by 2040, which differs by 2.6% from projection method 2 (assuming annual change 2006–2014) (see Additional file 2). To assess the impact of data collection period on our projections, we compared the palliative care estimate reached using 2014–2015 population data with 2014 death registry data, to the same estimate using 2013–2014 population data with 2014 death registry data. We found that using the later period reduced the palliative care need estimate by 0.5%.

## Discussion

This study projects, for the first time, an estimate of palliative care need 25 years into the future, showing that between 25% and 47% more people may need palliative care by 2040 in England and Wales. The projected rise in deaths from chronic diseases, in addition to increasing overall deaths, and more deaths occurring at older ages, will drive growth in palliative care need that is much greater than previously expected. Our projection of mortality for major disease groups further indicates that the dominant illnesses accounting for the growth in palliative care need will be dementia and cancer.

It is as yet unclear exactly how health professionals are to meet the increased population palliative care need. If all are to receive palliative care from specialist teams, a massive increase in training of specialist nurses and physicians is needed now. On the other hand, generalists may continue to provide the majority of end-of-life care, especially in community settings, with support from specialist teams for patients with more complex needs [37, 38]. To achieve this, we will need more staff, but also additional training in palliative care for non-specialist healthcare professionals currently providing care at the end of life, including oncologists, general practitioners, elderly care physicians and community nurses, amongst others. It takes at least 9 years to train a community geriatrician, and so workforce planners need to act now [39]. Further research into education is also needed to ensure we provide optimal healthcare professional training in palliative and end-of-life care.

These results are important to those planning health services because they indicate that the high proportion of resources already required in the last year of life [11, 13, 40] will rise markedly. If palliative care need increases as these projections indicate, this amounts to a huge challenge for both generalist and specialist palliative care services, and therefore supports an argument for changes in models of palliative care delivery. Specialist palliative care services still mainly treat patients with cancer [41], and we project that this need will grow, particularly given the high prevalence of pain and other symptoms in advanced cancer. However, the unprecedented increase in deaths from dementia means that a change in focus towards people with dementia will also be required.

Since the disease trajectory of dementia is both prolonged and unpredictable [42], specialist palliative care services may need to care for individual patients for longer periods of time and potentially provide support for patients at several time points, which may be unsustainable with current care models. Integration of palliative care into mainstream healthcare provision may be a way to support co-ordinated care for these patients, focusing particularly on those with complex needs [37]. Another approach is to develop short-term palliative care interventions at key time points within the disease trajectory [43, 44]. A third key approach is to focus efforts earlier in the disease course. Interventions such as advance care planning may allow needs to be met in line with patient preferences as illness progresses [45]. To achieve this, prospective identification of advanced disease and potential palliative care need is vital. Further work is needed to identify who may benefit most from palliative care [46] and to understand the stability of preferences for patients facing a prolonged and uncertain illness trajectory [47].

In this analysis, we have used several years of high quality, whole-population data to analyse mortality trends. The ICD-10-based palliative care estimate uses an evidence-based code filter which has been developed iteratively over several years, and has been tested internationally [21]. In reality, the situation is more complex than our estimates suggest, since the large majority of people who die, especially those dying at older ages, will have more than one illness before they die [48, 49]. Multimorbidity increases with age [48–50], which is particularly relevant because far more deaths are projected to occur at older ages by 2040. We cannot account for multimorbidity in this cause-of-death-based estimate, but it is safe to assume that, as well as the condition recorded as the ‘main’ cause of death, many people in our estimate will have had other comorbid conditions. This means that the complexity of palliative care need is likely to grow due to the high symptom burden [51], complex healthcare needs [52], and high hospitalisation rates [53] of patients with multimorbidity. Our projections may therefore underestimate overall palliative care need. Future plans for end-of-life care must account for the rising healthcare use of older adults with comorbidity [49], and health services must adapt from the current tendency to focus care on single organ disease, to more co-ordinated, person-focused care. Appropriate integrated models of care, including integrated short-term specialist palliative care services, may help to improve care co-ordination and allow care delivery in line with individual preferences [43, 54, 55].

Our projections should not be interpreted as a forecast of what will happen. Rather, they are projections of what may occur if recent trends in cause of death continue. Other approaches, such as that of Lee and Carter [36], have been used elsewhere to provide robust projections of mortality. However, regular coding changes in mortality datasets mean that only a short base period is available for analysis, limiting the value of these approaches to project disease-specific mortality [56]. Nevertheless, our sensitivity analysis using the Lee–Carter approach produced comparable results to the main forecasts. Our aggregate model uses recent trends to project future levels of need. Because of this, we cannot account for potential future changes in medical treatments or patient outcomes, such as the impact of a rise in obesity, or advances in cancer treatment [8]. This is particularly important for dementia – whilst current trends suggest an increase in prevalence of dementia to two million people in the UK by 2051 [57], some evidence suggests dementia prevalence may soon start to fall due to improved management of risk factors [58]. If dementia prevalence does fall, the rise in dementia deaths will be smaller than recent trends suggest. Nevertheless, more people will live with chronic illness and multimorbidity as life expectancy rises [48]. We can therefore be confident that, even if dementia prevalence falls, population palliative care need will rise markedly.

We have limited our projections to 2040 because of the increased uncertainty in mortality projections after this point. However, population data indicates that, whilst deaths in each age group over 65 are increasing in number up to 2040, deaths as a proportion of the population still alive in each age group over 65 are falling [7]. This means that, by 2040, many more people will be living over the age of 65. What is certain is that all of these people will eventually die, meaning that population palliative care need in older people will increase beyond 2040.

This study shares the limitations of all analyses using death registry data in that it relies on accurate completion of death certificates. Some causes of death are inconsistently recorded [59, 60] and this can lead to over- or under-reporting. These errors tend to be quite specific (e.g. colon cancer vs. rectal cancer [60]), so our choice of broader disease groups will reduce errors. Using death registry data also means that we can only estimate palliative care need at the end of life. Whilst the majority of palliative care needs do occur in the last months of life, palliative care needs are increasingly recognised earlier in the disease course, and such needs would not be captured by our estimate.

## Conclusions

This study builds on existing population palliative care estimates by investigating future palliative care need. Our analysis indicates that palliative care need will grow far more over the next 25 years than previously expected. The rise in deaths from chronic illnesses likely to require palliative care means that need will grow out of proportion to expected demographic changes. Many high-income countries will undergo similar population and disease changes and so can expect comparable increases in palliative care need. Current models of palliative care must adapt to these projected changes, and greater focus on non-specialist health professional education is needed. In particular, we must prepare for the growth in dementia and cancer if we are to provide appropriate care to people dying in the future.

## Additional files

Additional file 1: Technical considerations for palliative care estimates and projections. This file contains discussion of technical areas considered in the undertaking of this analysis. (DOCX 16 kb)

Additional file 2: Lee–Carter projection of palliative care need. This file presents brief details of a sensitivity analysis undertaken in relation to projection method 2 (assuming annual change). (DOCX 15 kb)

Additional file 3: Additional references. This file contains additional references cited in Additional files 1 and 2. (DOCX 10 kb)

## Abbreviations

ICD-10: International Classification of Disease version 10
ONS: Office of National Statistics
