# Supplementary material for: How many people will need palliative care in 2040? Past trends, future projections and implications for services
Source: BMC Med. 2017 May 18;15:102. doi: 10.1186/s12916-017-0860-2 (PMC5436458; doi:10.1186/s12916-017-0860-2)
Supplement: Supplementary file 1 — Technical considerations for palliative care estimates and projections. This file contains discussion of technical areas considered in the undertaking of this analysis. (DOCX 16 kb) [file 12916_2017_860_MOESM1_ESM.docx]

**Additional file 1:**

**Technical considerations for palliative care estimate and projection methodology**

1. *Combining ONS death registry and mortality projections data:*

Our analysis required that we combine ONS death registry and mortality projections data. When combining these data sources, we needed to account for the time-period over which the data were collected. Mortality data use deaths *registered* in a calendar year, rather than deaths *occurring* in a calendar year. Due to registration delays, approximately 4% of deaths registered in a calendar year occurred in the previous year.[Supplementary reference 1] Population and mortality projections, on the other hand, are a mid-year estimate based on change over the previous 12 months. We used population data for June 2013- June 2014 alongside death registry data for January - December 2014, and so on. To assess the impact of data collection period on our projections, we undertook sensitivity analysis. In this analysis we compared the palliative care estimate reached using 2014-15 population data with 2014 death registry data, to the same estimate using 2013-14 population data with 2014 death registry data.

1. *Accounting for ONS death registry coding changes in method 2 (assuming annual change):*

Our projection method 2 (assuming annual change) uses a base period of death registry data going back to 2006. Any changes in coding practice over this time period could potentially affect the annual change that forms the basis of this projection. We therefore considered carefully any changes in ONS coding of death registry across the base period.

ONS death registry data is based on ICD10 coding of the information provided on the death certificate. A ‘main’ cause of death is produced based on this information. The coding system was updated in 2011, meaning that deaths occurring in 2010 were coded differently to those in 2011. This coding change is described in detail elsewhere,[29] but the main areas affected are deaths occurring due to some cardiovascular diseases, and dementia. The most important change for this analysis is that many deaths previously recorded under I67.9 – ‘Cerebrovascular disease, unspecified’, were subsequently coded within F01 – ‘Vascular Dementia.’

Since both of these codes fall within our overall palliative care estimate, this is relatively unaffected. We nevertheless projected a palliative care need estimate based on 2006 to 2014 trend as well as a 2011 to 2014 trend (figure 1) to assess this. We found a difference of 2.5% between these estimates at 2040.

However this change does affect the disease specific projections. Using data from 2006 to 2014 for the disease specific projections provides a longer base trend in mortality, so we applied this method where possible for each disease group. However, the 2011 ICD10 coding change meant that some deaths came out of our ‘Other’ group in 2011 and moved into the ‘Dementia’ group. This can be seen in the change in number of deaths from 2010 (pre-coding change) to 2011 in these disease groups: between 2010 and 2011 Other deaths dropped by 8,062 and Dementia deaths increased by 13,175. Using a base period this time period would result in an unrealistic rise in Dementia deaths, and an unrealistic reduction in Other deaths if the trend were projected. Therefore, for the Other and Dementia groups, we used a shorter trend from 2011 – 2014 for our projection.

1. *Imputation of negative values:*

In a small number of cases, projections using method 2 (assuming annual change) resulted in negative numbers of deaths in a given gender and age bracket for one of the disease groups. This is related to the extrapolation methodology. Because this is unrealistic, we replaced the negative values with zero. This imputation has minimal impact on our findings; it resulted in a difference of 458/628,659 deaths in 2040 (error of 0.07%).

1. *Sensitivity analysis for projection method 2 (assuming annual change):*

Projection method 2 (assuming annual change) takes the mean annual change in mortality for the included ICD10 codes and assumes this mean annual change will continue to occur for each year of the projection. This is a simple extrapolation, and other methods exist which use different assumptions. We therefore undertook sensitivity analysis with an alternative projection methodology to test our approach. We used the Lee-Carter method, which has more commonly been applied to population data with a long base period,[36] and also applied the Coale-Guo extrapolation to undertake population age smoothing above age 80.[supplementary reference 2] This sensitivity analysis resulted in a 2.6% difference from our main methodology, as reported in the results section.
