# Supplementary material for: How many people will need palliative care in 2040? Past trends, future projections and implications for services
Source: BMC Med. 2017 May 18;15:102. doi: 10.1186/s12916-017-0860-2 (PMC5436458; doi:10.1186/s12916-017-0860-2)
Supplement: Supplementary file 2 — Lee–Carter projection of palliative care need. This file presents brief details of a sensitivity analysis undertaken in relation to projection method 2 (assuming annual change). (DOCX 15 kb) [file 12916_2017_860_MOESM2_ESM.docx]

**Additional file 2:** Lee-Carter projection of palliative care need, calculated using the LCFit web platform.[supplementary reference 3]

| Age group | Crude mortality rates 2010- 14  (for palliative care ICD10 codes as per table 1 main manuscript) | | | | | Projected Mortality Rate 2040 (palliative care ICD10 codes) | Projected population of England & Wales mid 2040 | Projected deaths requiring palliative care 2040 |
| --- | --- | --- | --- | --- | --- | --- | --- | --- |
|  | 2010 | 2011 | 2012 | 2013 | 2014 |  |  |  |
| 0-4 | 0.000047 | 0.000043 | 0.000051 | 0.000049 | 0.000037 | 0.000002 | 3723349 | 7 |
| 5-9 | 0.000030 | 0.000040 | 0.000031 | 0.000031 | 0.000038 | 0.000593 | 3703491 | 2196 |
| 10-14 | 0.000035 | 0.000042 | 0.000031 | 0.000032 | 0.000031 | 0.000028 | 3752404 | 105 |
| 15-19 | 0.000059 | 0.000054 | 0.000053 | 0.000047 | 0.000048 | 0.000015 | 3857782 | 58 |
| 20-24 | 0.000076 | 0.000077 | 0.000075 | 0.000066 | 0.000074 | 0.000092 | 4030625 | 371 |
| 20-29 | 0.000133 | 0.000131 | 0.000130 | 0.000128 | 0.000120 | 0.00005 | 4258068 | 213 |
| 30-34 | 0.000258 | 0.000231 | 0.000225 | 0.000228 | 0.000249 | 0.000349 | 4196149 | 1464 |
| 35-39 | 0.000499 | 0.000475 | 0.000418 | 0.000448 | 0.000384 | 0.000068 | 3776529 | 257 |
| 40-44 | 0.000933 | 0.000869 | 0.000850 | 0.000850 | 0.000799 | 0.000274 | 3932990 | 1078 |
| 45-49 | 0.001635 | 0.001532 | 0.001507 | 0.001525 | 0.001570 | 0.00156 | 4128742 | 6441 |
| 50-54 | 0.002883 | 0.002741 | 0.002601 | 0.002610 | 0.002958 | 0.007079 | 4007562 | 28370 |
| 55-59 | 0.004994 | 0.004855 | 0.004599 | 0.004586 | 0.004193 | 0.001197 | 3822377 | 4575 |
| 60-64 | 0.008035 | 0.007791 | 0.007680 | 0.007789 | 0.007607 | 0.005395 | 3482796 | 18790 |
| 65-69 | 0.013038 | 0.012310 | 0.011961 | 0.011790 | 0.011699 | 0.006591 | 3548593 | 23389 |
| 70-74 | 0.021015 | 0.020750 | 0.020275 | 0.019952 | 0.019242 | 0.010283 | 3619068 | 37215 |
| 75-79 | 0.034319 | 0.032684 | 0.032502 | 0.032081 | 0.031380 | 0.01737 | 3278584 | 56949 |
| 80-84 | 0.057859 | 0.055718 | 0.056407 | 0.055878 | 0.054581 | 0.03006 | 2465054 | 74100 |
| 85-89 | 0.098106 | 0.093757 | 0.096157 | 0.095458 | 0.094506 | 0.053292 | 1702260 | 90717 |
| 90-94 | 0.155313* | 0.156959* | 0.163364* | 0.164618* | 0.162238* | 0.09679 | 1108375 | 107280 |
| 95-99 |  |  |  |  |  | ^#^0.180087 | 375743 | 67666 |
| 100-104 |  |  |  |  |  | ^#^0.343262 | 75348 | 25864 |
| 105+ |  |  |  |  |  | ^#^0.670283 | 6031 | 4042 |
|  |  |  |  |  |  |  | **66851920** | **551146** |

* For base period mortality data, a single rate for ages 90+ is used

^#^ For Lee-Carter projection model, population age smoothing is applied above age 80 using the Coale-Guo extrapolation method.[supplementary reference 2]
