# Supplementary material for: How many people will need palliative care in 2040? Past trends, future projections and implications for services
Source: BMC Med. 2017 May 18;15:102. doi: 10.1186/s12916-017-0860-2 (PMC5436458; doi:10.1186/s12916-017-0860-2)
Supplement: Supplementary file 3 — Additional references. This file contains additional references cited in Additional files 1 and 2. (DOCX 10 kb) [file 12916_2017_860_MOESM3_ESM.docx]

**Additional file 3. Additional references:**

Supplementary reference 1. Office for National Statistics: **Mortality Statistics: Metadata**. 2015. [www.ons.gov.uk/ons/guide-method/user-guidance/health-and-life-events/mortality-metadata.pdf] Accessed 14th November 2016.

Supplementary reference 2. Renshaw A and Haberman S. **Lee–Carter mortality forecasting: a parallel generalized linear modelling approach for England and Wales mortality projections.** *Journal of the Royal Statistical Society:* Series C (Applied Statistics). 2003; 52: 119-37.

Supplementary reference 3. Sprague, W. Webb (2009) **LCFIT** (web based software for estimating the Lee-Carter method for modelling and forecasting mortality, with extensions to Coherent Forecasts of Li and Lee, and Lee-Carter method with sparse and incomplete data)" Accessed at <http://lcfit.demog.berkeley.edu/> on 23/01/2017
